# The Significance of Experiences of Nature for People with Parkinson’s Disease, with Special Focus on Freezing of Gait—The Necessity for a Biophilic Environment. A Multi-Method Single Subject Study

**DOI:** 10.3390/ijerph120707274

**Published:** 2015-06-29

**Authors:** Johan Ottosson, Lillian Lavesson, Stefan Pinzke, Patrik Grahn

**Affiliations:** The Department of Work Science, Business Economics and Environmental Psychology, Swedish University of Agricultural Sciences, P.O. Box 88, SE-230 53 Alnarp, Sweden; E-Mails: Johan.Ottosson@slu.se (J.O.); Lillian.Lavesson@slu.se (L.L.); Stefan.Pinzke@slu.se (S.P.)

**Keywords:** natural environments, biophilia, perception, instincts, supporting environments, phobic reactions, attention

## Abstract

Freezing of Gait (FOG) is a common condition in people with Parkinson’s disease (PD). FOG entails suddenly experiencing difficulties moving or feeling that one’s feet are as glued to the ground. It is triggered, e.g., when passing through doorways. Earlier studies suggest that being in natural environments affects FOG in a positive way. Five subjects were recruited to serve as five single subject cases. We used interviews, observations, questionnaires and collected gait pattern data with aid of an accelerometer. A special designed outdoor setting was used, where we investigated whether passing through hedge openings with or without built elements triggered FOG. We found that no one experienced a FOG reaction when they passed through hedge openings without built elements. However, FOG was triggered when a doorframe was inserted into a hedge opening, and/or when peripheral vision was blocked. We interpret the results such that the doorframe triggered a phobic reflex, causing a freezing reaction. Passing through hedge openings does not trigger FOG, which we interpret as a biophilic reaction. Our results, if repeated in future studies, may have significance to everyday lives of PD patients, who could get a simpler life by consciously prioritizing stays in natural surroundings.

## 1. Introduction

Parkinson’s disease (PD) is the second most common neurodegenerative disorder after Alzheimer’s disease, and affects approximately seven million people globally [1]. Each year, between 4 and 20 individuals per 100,000 fall ill from PD [2]. In Sweden, approximately 20,000 people have been diagnosed, and about 1500 more are diagnosed annually [3]. Typical disease symptoms include hypokinesia (akinesia, slowness, and impaired mobility), muscle rigidity (involuntary muscle tension), tremor (shaking/trembling of the limbs), and simultaneously diminished capacity [4]. Rigidity and akinesia mean that it can be difficult to keep one’s balance.

More than half of those in the advanced stages of PD develop “freezing” [5] which is characterized by a sudden difficulty/inability to walk: Their movement freezes (Freezing of Gait, FOG) [6]. People who have developed FOG can suddenly experience that their feet are glued to the ground, and find it difficult to maintain their balance. FOG is characterized by a sudden inability to initiate or continue walking, especially when changing direction in stressful time-limited situations, at an entrance and through narrow spaces such as doorways [6,7,8,9,10,11]. FOG has been interpreted as motor blocking in limbs. However, research now suggests that other factors, such as the influence of different memory systems in the brain, may contribute [12,13]. FOG has also been shown to be associated with anxiety, depression, stress, and pain [6,14,15,16].

In an intervention study carried out in the Swedish mountains with 12 people with PD, Sunvisson *et al.* [17] found that the motor performance of the subjects improved. This intervention was carried out for three consecutive years. In a qualitative follow-up study, subjects claimed that by walking through marshes and on moors, they regained their body rhythms; they relaxed and experienced a harmony not only between body and mind, but also with Nature. They experienced an absence of stress [18]. Usually, the disease forced them to focus on their body to be able to move. In Nature, this focus became transformed and was directed outward [18]. Could it be that natural areas do not trigger FOG to the same extent as built areas?

Nature-assisted interventions have a long tradition in health care, and interest has increased in recent years, both regarding research and applications in the field. Annerstedt and Währborg [19] surveyed 38 carefully selected and well-conducted scientific studies on the impact of nature-assisted interventions, and they concluded that this kind of intervention leads to significant improvements for various outcomes in different diagnoses. In the past few years alone, several well-conducted Randomized Controlled Trials (RCT) and studies of a similar design have been published. Nature-assisted interventions have often proved to be significantly better than the usual treatments. This is valid for several different types of diseases, and measurable effects relate to reduced symptoms of illness e.g., [20,21,22,23], and increased levels of function [21,24,25]. Significant differences in RCT studies concerning physiological markers have also been registered [26,27], as well as a significant reduction—Compared with a matched control group—Regarding health care consumption [28]. There are also studies on neurological diseases such as stroke showing effects concerning the reduction of self-reported symptoms and an increased level of function [29].

Human beings communicate with their environment via perception. In recent years, the integration of senses and how sensory information is interpreted by the brain has attracted increasing attention. This applies to sight, hearing and, not least, to senses important for movement such as balance, proprioceptive senses, and the vestibular sense [30]. Research on how the brain interprets sensory input suggests that our perception registers our surroundings based on threat or safety. Natural environments can be instinctively interpreted in the form of “danger” or “no danger” [31]. This occurs quickly and unconsciously. The body reacts by increasing or reducing levels of stress [32,33]. During this interpretation process, innate reflexes may play a decisive role in how we react [34,35,36].

Furthermore, research suggests that our attention can be divided into types. One, “directed attention” or “voluntary attention”, is used to a large extent in e.g., offices, and involves our executive functions sorting information, assessing, evaluating, prioritizing, and executing what is needed. This type of attention requires a great deal of effort. The other is “fascination” or “involuntary attention”—a more spontaneous type of attention which is used, e.g., to keep track of and discover things in the environment, such as rustling in the bushes or a flashing light [37,38]. Hard fascination occupies our full attention, and leaves no scope for our own reflections, while soft fascination is associated with secure environments and requires very little effort [37]. In nature, we rely on the spontaneous attention that seems to easily decode our surroundings as entailing “hard” or “soft” fascination. However, when in a built environment, we have to focus, choose, sort, and then interpret the impressions we receive [37,39]. Hence, one assumption could be that decoding a built environment as “no danger” is not done in a quick, unconscious way.

Freezing is very difficult to measure in experimental settings. However, several studies indicate that FOG reactions can be associated with e.g., stride length and walking speed variability prior to a freezing episode [40]. This has also been shown in a study by Almeida and Lebold [11], where subjects passed through doorways of various widths. The narrowest doorway had the greatest effect on walking behavior. The authors assumed that FOG has motoric origins but that the spatial experience is also of significance [11]. Their conclusion was that FOG can be brought on by visual input and underlying perceptual mechanisms interfering with movement planning. This means, among other things, that visual stimuli influence whether the FOG reaction is triggered or not [11].

Our hypothesis is that the natural environment does not trigger FOG reactions to the same extent as the built environment. We had two objectives in the study: the first aim was to study if the hypothesis is correct, through a series of experiments, while the second aim was to create possible explanations and theories related to findings. Thus the research question were:

Can FOG be triggered by walking through narrow passages in Nature?
Can FOG be triggered to the same extent in passages in natural contexts when adding a built element?
Can FOG be triggered when the peripheral visual field is limited?
Can our findings be explained by or generate any theories or hypotheses?

We were interested in observing the FOG reaction throughout the whole experiment and particularly when walking through a passage. This was to study how FOG reactions develop in an experimental setting.

## 2. Experimental Section 

Given the complexity of the issue and that the reaction is difficult to assess and measure, we chose to test our hypothesis by using a multi-method approach which was both quantitative and qualitative, but mainly qualitative.

The clinical picture of Parkinson’s disease is that most patients have several features in common, but there are considerable individual differences. In addition, the disease evolves constantly, as does medication and treatment for the individual patient [6,41]. A description concerning an individual patient’s status can therefore be regarded as a sort of “instant statement”. Hence, “single-subject research design” has been used, because it is sensitive to individual differences and produces both descriptive and correlation data, and can be used to detect causal connections [42,43,44]. It has been of principal importance in this study to be able to collect subjects' experiences of FOG which can be attributed to an experimental situation in a real environment. Experience sampling methodology (ESM) [45] is an approach which involves gathering from subjects relevant data associated with specific episodes—such as, in this case, regarding experiences of FOG associated with specific places or events. Self-reported data is included in ESM, as is self-observation [46,47], which adds valuable self-perceived knowledge of collected data. A particular type of ESM, Ecological Momentary Assessment [48], is especially designed to focus on micro processes that influence behavior in real-world contexts; this resulted in us choosing this type of assessment. Because Parkinson’s disease manifests itself in different ways for men and women [49,50], even concerning the incidence of FOG [51], we chose in this study to focus on men. Since we wished to have rich qualitative descriptions of the phenomenon FOG in this experimental situation where FOG was provoked, it led to the number of cases must be limited [52,53,54]. In order to present a nuanced result that describes the phenomenon, we chose to limit the study to five participants.

### 2.1. Subjects

The subjects in the test were five persons, recruited by the Swedish Parkinson Association. Inclusion criteria: (1) Male participants; (2) Clinically typical PD as confirmed by diagnosis and known to be responsive to antiparkinson medication; (3) Confirmed to be experiencing FOG at the time of the test and experiencing FOG reactions in door passages. They were five men, 73, 69, 64, 60, and 60 years old. The study was approved by ethical committee: The participants could voluntarily participate in the study on own terms. All subjects were informed about the study. They continued to take their medication as usual. However, we wanted to measure the subjects when they experienced FOG, which often occurs when the medication level is low. When the medication level is low, participants often end up in an off-phase. Owing to that, they become more sensitive to the surroundings and hence more often experience FOG-reactions. We asked for written consensus before the participants voluntarily entered the study. The participants could at any point end the study without further explanations. All gave written consent. All subjects came with their spouse, who helped and supported them. 

### 2.2. Venue and Experimental Conditions

**Figure 1:**
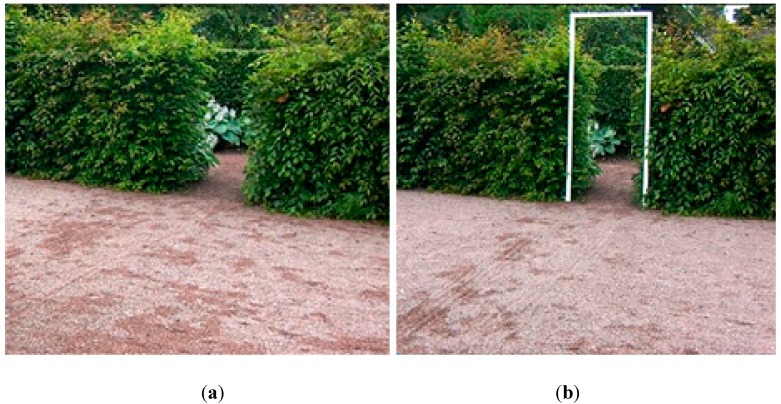
(**a**) Passageway through only the hedge opening; (**b**) and through the hedge opening with the white doorframe.

The study was conducted at the Alnarp Rehabilitation Garden, situated at the Swedish University of Agricultural Sciences, Alnarp Campus, 15 km from Malmö in the south of Sweden [55]. This garden has several 2 m high, dense hornbeam (*Carpinus betulus*) hedges, with openings specially designed for the test (Figure 1a).

The experimental setup of the study and the quantitative measurements has, to some extent, been based on Almeida and Lebold [11]. To indicate a built environment, a white doorframe was placed in the hedge opening. However, the doorframe has a horizontal bar on top, while the passageway through the hedge opening lacks a horizontal upper restriction. No other changes were made. The approach to the hedge opening was 4 m long. During the test, movement patterns (such as cadence, stride, and acceleration) were measured using an accelerometer. For analysis and documentation purposes, the entire walking sequence was videotaped (Figure 1b).

Weather conditions were recorded (temperature, wind, sunny, or overcast). High temperatures (+25 degrees Celsius and above, especially with high humidity) were known to affect the subjects negatively (personal communication from Gun Lindahl, Parkinson nurse, 3 November 2013).

### 2.3. Quantitative Data

#### 2.3.1. Questionnaire

Self-estimations of the experience of FOG were measured before and immediately after each whole test round, using a ten-point Likert scale with the end points 0 (no FOG) and 10 (maximal FOG). The questionnaire contained two questions: “Estimate your FOG-reactions throughout the test”, and: “Estimate your FOG-reactions when passing through the opening”.

#### 2.3.2. Accelerometer

A three-axis accelerometer (Model X6-2 from Gulf Coast Data Concepts, LLC, Waveland, MS, USA) was used to register acceleration in three directions with a 40 Hz sampling frequency. The accelerometer was placed in the subject’s sock at the Achilles tendon of the left heel. 

**Figure 2:**
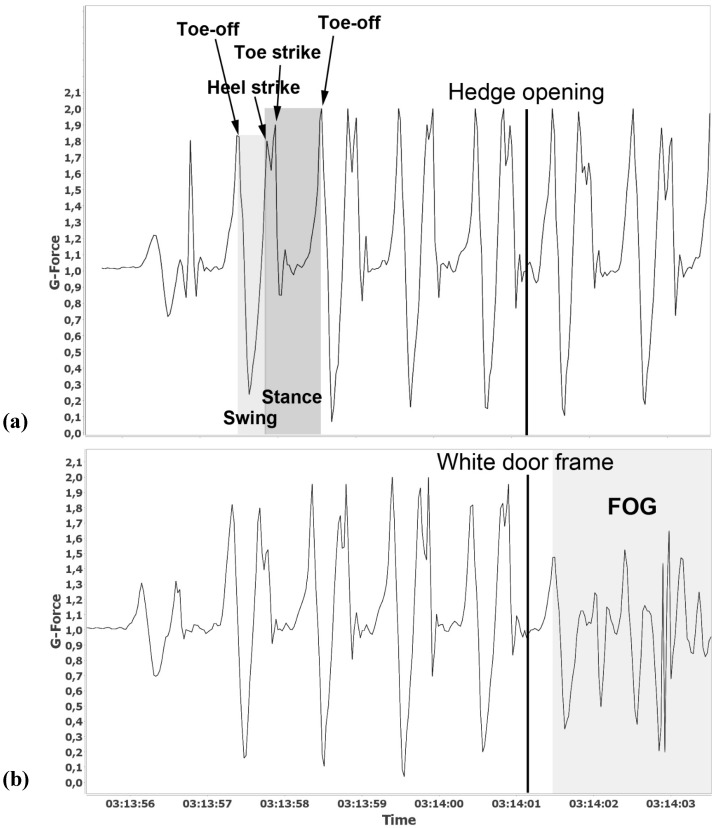
Accelerometer measurements. Example of patterns when passing through the hedge opening (**a**) without the white doorframe and (**b**) with the white doorframe. The height of the amplitudes corresponds to the acceleration of the step.

To analyze gait patterns, the summed and normalized wave form of data for the three directions was studied. A step cycle contains a swing phase and a stance phase. The swing phase begins when the foot leaves the ground (toe-off) and ends when it touches the ground again (heel strike and toe strike); then the stance phase begins, and ends at the next toe-off. Cadence, stride, and step-speed can be calculated by identifying the high peak amplitudes for toe-off, heel strike, and next toe-off on the wave form. The height of the amplitudes corresponds to the acceleration of the step (Figure 2). Printouts of accelerometer measurements were made. The patterns were assessed as follows: No deviations, deviations (change in acceleration or step length) and strong deviations (both change in acceleration and step length).

### 2.4. Qualitative Data

#### 2.4.1. Interviews

In order to describe the subjects’ experiences of FOG, semi-structured interviews [56] were conducted. Before the trial started, subjects had to describe their situation in everyday life, how it is affected by FOG. The interviews were performed in Swedish, face-to-face. The three first authors performed the interviews. Each interview lasted about 40 minutes. The interviews were documented using handwritten notes and analyzed regarding important concepts using qualitative content analysis [57]. The process of analysis was conducted by the four authors together.

#### 2.4.2. Observations

Observations were conducted both by a licensed physical therapist during the experiments and, as an additional assessment, by subsequent review of the video by all authors together. The observations focused on estimating posture, movement and walking patterns during the tests. An assessment was carried out regarding the subjects’ walking patterns during the tests and FOG reactions in the passages. Not observable, observable, strong, and very strong were used.

### 2.5. Pre-Test Procedure

Each subject was instructed to turn 360 degrees to insure that they experienced FOG reactions [58]. All of the subjects showed clear symptoms of FOG.

### 2.6. Test Procedure

Subjects were instructed to walk through the hedge openings. We did not say anything to the subjects about our expectations, just asked them to walk through the openings as they usually do.

The subjects started four meters in front of the opening. They were instructed to close their eyes and count to ten before opening their eyes and starting to walk. The procedure took about an hour to complete. Tests were conducted in full daylight. Each subject had their own day of testing.

Test Round: Walking test through a hedge opening with and without a built element.

(i) Walking test through a hedge opening 80 cm wide. This was repeated three times.
(ii) A white doorframe (Figure 1(b)) was placed in the hedge opening. Its inner measurements were 80 cm wide by 210 cm high. The second walking test included the frame, highly visible in the opening; all other aspects were the same. This was repeated three times.
(iii) Walking test through the hedge opening without the frame. This was repeated three times.

Test Round: Continued attempts with two subjects.

Continued attempts were made with the two subjects who were showing the strongest FOG reactions when turning 360 degrees. One of them, number one, exhibited the strongest FOG reaction, whereas the other, number two, showed no FOG reaction at all at the passage with the door-frame.

Test round: Walking test through a hedge-opening with door-frame.

(i) Hedge opening with a white doorframe which was placed in the hedge opening. It measured 80 cm wide by 210 cm high. The walking test included the frame, highly visible in the opening; all other aspects were the same. This was repeated three times.
(ii) Hedge opening with a white doorframe. This was repeated three times.
(iii) Hedge opening with a white doorframe. This was repeated three times.

Test Round: Continued attempts with subject number 1, with the strongest FOG reaction in door- frame.

Continued attempts were made with subject number one who exhibited the strongest FOG reaction.

Test Round 1: Hedge opening with a narrow doorframe.

(i) Walking test through a hedge opening 67.5 cm wide. This was repeated three times.
(ii) A white doorframe was placed in the hedge opening. It measured 67.5 cm wide by 210 cm high. The second walking test included the frame, highly visible in the opening; all other aspects were the same. This was repeated three times.
(iii) Walking test through the hedge opening without the frame. This was repeated three times.

Test Round 2: Paper cylinder.

(i) Walking test through a hedge opening 67.5 cm wide. This was repeated three times.
(ii) Walking test through a hedge opening 67.5 cm wide while limiting the subject’s field of peripheral vision using a paper cylinder to blinker him. This was repeated three times.
(iii) Walking test through the hedge opening without a paper cylinder to blinker the subject. This was repeated three times.

Test Round 3: Door-frame and paper cylinder.

(i) Walking test through a hedge opening 67.5 cm wide. This was repeated three times.
(ii) A white doorframe was placed in the hedge opening. It measured 67.5 cm wide by 210 cm high. The second walking test included the frame, and limiting the subject’s field of peripheral vision using a paper cylinder to blinker him. This was repeated three times.
(iii) Walking test through the hedge opening without the frame or the paper cylinder. This was repeated three times.

## 3. Results

### 3.1. Interviews before the Test

Subject number one feels an indefinable unease about passing through doors because he often experiences the FOG reaction. He usually skips or jumps with both feet together with the aid of the walker to get out of FOG. Subject number one feels strong FOG in everyday situations. He moves more easily outdoors than indoors. See Table 1.

**Table 1:** Results from the first interview in a summary overview.

Subject	1	2	3	4	5
Cognitive experience in connection with FOG	Feeling of insecurity and discomfort	Having trouble thinking and expressing himself	Unfocused, having difficulties thinking and expressing himself, having difficulties remembering	Problems concentrating when tired, having trouble keeping his eyes open	Difficulties with speech; slurs his words. Better when in a good mood.
Physical experience in connection with FOG	Experiencing strong locking	Stiff and locked, finding it difficult to control his body, getting uncontrolled body movements	Stiff, difficulties lifting legs	Noticing a distinct stiffness, having difficulties with balance. Falling sometimes	Distinct stiffness, and aches
Strategy	Deliberately uses unusual behavior to walk, “unnatural”, for example, walks backwards, skips or jump with both feet together.	Walks with small, skipping steps, which were more common in the past, now used more rarely	Skips, concentrates on lifting legs	Dances. Eyes closed upon passage	Skips, and turns in his elbow upon passage
Differences in walking, outdoors/indoors	Moves more easily outdoors	Doesn’t know	Moves more easily outdoors	Moves more easily outdoors	Missing data

Subject number two says that it is “getting harder to think and express himself when experiencing FOG”. Physically, he notices that he feels stiff and that his body becomes more locked, but he also notices involuntary body movements such as head movement—“it feels like your head is dangling”. It is as if he loses control of his body. Previously, he used skipping steps as a strategy, now this is increasingly rare.

Subject number three knows that he cannot think clearly, remembers things poorly, and that he sometimes is unable to express himself. He says that he “has to concentrate when he walks” when “it feels like he otherwise cannot lift his legs”. According to a relative, “sometimes he has to skip, at any time”, to cope with FOG. In contrast, for example, to when he mows the lawn, when he gets no FOG. He mows the lawn using a hand mower. The family says that the subject performs worse indoors than outdoors when he is walking outdoor on the lawn. Relatives say that if the tests were carried out earlier in the morning, the subject “would have had severe FOG in the doorway”.

Subject number four feels stiffness, mostly in his left leg when he experiences FOG. He also knows that his balance is affected. He says he has difficulty keeping his eyes open. He often experiences FOG, and FOG is strong at night and early in the mornings. It helps to dance. When his wife sings a waltz, the subject can make his way through doorways and break lockings. He has found that if he closes his eyes when going through doorways, he feels less FOG. He thinks he moves easier outdoors than indoors. The stiffness is strongest on his left side and balance is affected. He has fallen when experiencing FOG and he falls regularly, especially when he is carrying something in his hands, such as a tray—then he loses his balance more easily.

Subject number five’s biggest problems are stiffness in his legs and experiencing mental disturbances with slurred speech. The subject says that he can experience FOG at any time. He has to take his medication every two hours to avoid FOG. The subject’s wife says she has noticed that when there is something that the subject thinks is interesting and amusing, he experiences less FOG. He is very stiff in the morning and has found a way to get through doorways without getting FOG by hitting his elbow on the doorframe. A low level of medication manifests as the subject getting FOG more easily and a stiffness in his legs that becomes painful. In addition, there is an impact on his mental capacity with slurred speech. He has previously had a lot of cramp, but this has become much better with regular weight training at the gym. See Table 1.

### 3.2. Tests

Test Round: Walking test through a hedge opening with and without a built element.

The tests showed no FOG reaction when passing through the hedge openings without a doorframe. This was true for all subjects, was observed by the researchers and confirmed by accelerometer measurements, where no change in acceleration (amplitude of G-force) and stride (distance between two toe-offs) could be found (Figure 2a).

When passing through the hedge opening containing a doorframe, three subjects seemed to be unaffected while two had a clear reaction. One of those two showed a decrease in walking speed and took shorter steps. The other had an immediate and strong FOG reaction, without warning, with a change in stride length and speed. See Table 2. These two reactions were strong and clearly indicated in the accelerometer measurements, where FOG can be noted when acceleration (amplitude of G-force) and stride (distance between two toe-offs) change (Figure 2b). However, there was a slight deviation in accelerometer measurement for subject number five. See Table 2.

Test Round: Continued attempts with two subjects

The subjects filled out forms regarding self-estimation of their experiences of overall FOG on a Likert scale.

Subject number one rated his experiences of general FOG equally during the test rounds (6.00 for hedge openings and 6.05 for door-frames), see Table 3. However, despite similar self-estimates of general FOG, FOG was triggered only when passing through the hedge opening with the door-frame. The result of the previous test rounds was confirmed.

Subject number two’s self-estimates of general FOG at the various passages were, for passing through the hedge opening without a doorframe: 1.60 (mean), and passing through a door-frame: 6.60 (mean), but no FOG reaction was triggered. See Table 3.

**Table 2:** Results from tests, observations, and self-estimations in a summary overview.

Subject	1	2	3	4	5
**Weather conditions during the tests**	20 °C nice weather, cloudy with “rain in the air”	20 °C Sunny, variable with rainfall. High humidity. Light wind.	14 °C. Light rain before and after the tests. Light wind	8 °C Cloudy, no wind	8°C Cloudy, no wind
**360 degree turn**	Very strong FOG-reaction, jump with rollator	Very strong FOG-reaction, very stiff	Strong FOG-reaction, did not use his stick	Observable FOG-reaction, skipping steps	Observable FOG-reaction, turning on his heel
**Walking aids**	Walker	No aids	Stick	No aids	No aids
**Observed FOG, without doorframe**	Not observable	Not observable	Not observable	Not observable	Not observable
**Accelerometer, without doorframe**	No deviations	No deviations	No deviations	No deviations	No deviations
**Self-estimated FOG, Without doorframe (mean values)**	0.3	0	0	5.0	0
**Observed FOG, doorframe**	Very strong	Not observable	Not observable	Strong	Not observable
**Accelerometer, doorframe**	Strong deviations	No deviations	No deviations	Strong deviations	Deviations
**Self-estimated FOG at doorframe (mean values)**	7.3	0	0	6.0	6.0
**Self-estimated FOG Overall, during the test situation, (mean values)**	5.0	5.4	4.7	7.7	6.0
**Observed changes in walking patterns. Overall, during the test situation**	More constrained walking pattern; e.g., pulling up shoulders, change regarding the placement of one of his feet	Increased stiffness, less co-movement	Increased stiffness, stronger extra movements. Impaired coordination, does not use the walking cane anymore—Carries it instead	Increased stiffness, increased absence of co-movements	Increased rigidity, narrower step width, more bent knees and hip joints, increased posture leaning forward
**Observed changes in walking patterns when passing through the hedge opening with the doorframe**	Very strong FOG-reaction, his right foot turned inward, and there was a distinct slowness and stiffness (tardiness) in movements, and strong lockings.	No FOG reaction could be observed	No observable effect on walking behavior through the actual passages	A distinct FOG reaction and a clear observable decrease in stride length and walking speed before and during passing through the doorframe. Rigid movements, and observable that it becomes harder and harder to move as the FOG increases	No observable change in stride length, walking speed or posture when passing through the openings
**Differences between upper and lower body regarding FOG reactions. Overall, during the test**	None	FOG reactions only in the upper body	Apparent FOG reaction in the upper body, not regarding walking pattern, but the subject felt himself that his legs were affected	None	None, but he felt that his legs were affected
**Observed strategies to avoid FOG**	Jumping with the aid of the walker.	No change in the gait pattern can be observed. Marches. No tendency to FOG	Gait pattern changes due to increased stiffness, but no tendency to FOG	He closes his eyes	Increases his walking pace, half running

**Table 3:** Self-estimated overall FOG during continued tests with the two subjects showing the strongest FOG reaction when turning 360 degrees.

Subject	1	2
Self-estimated overall FOG when walking through the hedge opening (mean value)	6.00	1.60
FOG reaction when walking through the hedge opening	No	No
Self-estimated overall FOG when walking through the doorframe (mean value)	6.05	6.60
FOG reaction when walking through the doorframe	Yes	No

Test Round: Continued attempts with the subject with the strongest FOG reaction

The results from earlier tests were repeated and confirmed for subject number one. The tests were extended to limiting the subject’s peripheral visual field during the tests at the hedge openings with and without a doorframe.

In the test, the subject had strong FOG reactions when his field of vision was restricted using the cylinder, both when passing through the hedge opening with the doorframe and without the doorframe.

However, as tested before in previous rounds, no FOG reaction occurred when passing through the hedge opening without a cylinder. See Table 4.

**Table 4:** Self-estimated FOG and observable FOG reaction in test round with the subject with the strongest FOG reaction in doorframe.

Subject number one	Overall FOG	FOG when Passing through the Doorframe	FOG Reaction
Self-estimated FOG hedge opening (mean value)	8	0,5	No
Self-estimated FOG doorframe (mean value)	7	7	Yes
Self-estimated FOG hedge opening and paper cylinder (mean value)	7	4.9	Yes
Self-estimated FOG doorframe and paper cylinder (mean value)	8	7,5	Yes

### 3.3. Observations by a Physical Therapist

Subject number one walks with his head bent forward. He has an uneven walking rhythm, stiffer motion in his right leg compared with his left; walks on the outside of his right foot—with a whole heel strike on his left foot. His shoulders are significantly raised when starting the “doorframe test”, compared to starting the “hedge opening test”. He has a very strong FOG-reaction right at the doorframe. The freezing reaction comes very suddenly, without any warning. It was not preceded by any decrease in stride length or other observable physical reaction. When the FOG-reaction started, his right foot turned inward, and his movements became distinctly slow and stiff (tardiness) with strong lockings. The subject had to skip and jump with both feet together to get out of FOG with the support of a walker. See Table 2.

Subject number two walks leaning forward, looking down at the ground, peering. Co-movements in head and neck; his right arm follows the movements badly, but both arms are affected. His right arm is held in a backwards position. During the test, an increased stiffness could be observed in his arms, hands, and upper part of his body. With increased stiffness, involuntary movements of his head, hands, and arms were observed. His fingers and hands stayed bent, and an increased stiffness in his arms was observed. His legs were seemingly unaffected. It was evident that the subject stood with his body weight on his left leg when he was influenced of increased stiffness, rather than as previously with evenly distributed weight. No FOG reaction could be observed during the test. After the test, subject number two said he felt an increasing numbness from his knees up to his thighs during the test.

Subject number three walks with his head slightly bent forward and his gaze lowered. He has a kind of jerky walk, with observable involuntary co-movements in his left hand. He limps as his left leg moves forward in a pendula motion. The subject treads down on his entire left foot in a step but walks on the toes of his right foot. He is affected by inelasticity and rigidity and has a change in co-movements, especially on his left side. He walks with a cane in his right hand. Initially, it is used as support and relief, but when FOG increases, he carries the cane; this is probably caused by an inability to coordinate the cane with body movement—not that he did no longer needed the cane. No observable effect on walking behavior through either the green hedge opening or the white doorframe. After the test, subject number three experienced trouble with a so-called off-reaction during the experiment (medication too low), and felt increasingly unfocused. He experienced a strong mental, cognitive reaction during the experiment, and minor physical stiffness, and felt that he almost could not lift his legs.

Subject number four stands with noticeable stiffness in his body position, and with his feet far apart. Walks similarly with a “wide-track” walk, stiff, straight posture, and straight neck; much stiffness throughout his body, which gives uneven and jerky co-movements, an inflexible faltering walk. He holds his arms out from his body both when standing and walking. He does not bow his head as he walks. It is hard to see where his gaze is directed, but it is observable that he squints and eyes almost closed. He has a clear FOG-reaction when passing through the doorframe although he has his eyes closed when passing. There is a clearly observable decrease in stride length and walking speed before and while passing through the doorframe. His movements are rigid; and it is observable that it gets harder and harder for him to move his legs as the FOG increases. This was not observed when he passed through the hedge opening.

Subject number five walks slightly bent forward with his left hand, and sometimes both his hands, in his pockets and with slightly bent knees. His whole body leans slightly to the right. He holds both his arms close to his body and this gives an observable strong limitation of co-movements in his trunk, arms and shoulders. He walks with short, quick mincing and skipping steps. He walks with a small support area between his feet—Putting his feet down as on narrow parallel tracks—He does not walk in a line. He has his head in the same position all the time, but he shifts his gaze downwards when he walks. He has the same posture/position all the time—in all experiments. The subject almost runs to manage passing through—He almost bounces forward. No observable change in stride length, walking speed or posture when he passes through the hedge openings, either with or without the doorframe. He skips with fast, small steps, and no observable FOG-reaction. After the test, subject number five claimed that during the tests, his legs began to shake during the trial because of FOG and low medication. See Table 2.

## 4.Discussion

The discussion begins with the presentation of our main findings, and then follows a presentation of how these findings are interpreted, in relation to theories mainly within environmental psychology.

### 4.1. Main Findings

Our overall aim was to study if FOG-reactions are triggered in natural environments. We were interested in the FOG reaction throughout the whole experiment and especially when passing through openings. This was done to study how FOG reactions develop in an experimental setting.

One finding was that not one subject had a FOG reaction when they passed through the hedge opening, even though they all reacted when they walked through doorways inside buildings. The hedge opening was as wide as a normal doorway. Next, a built element in form of a doorframe was added into the hedge openings. No other changes were made. All subjects reported self-estimated FOG-reactions throughout the trial. Two subjects had clear FOG reactions in the opening when the doorframe had been added to the hedge opening. This was proved, both by a strong measurable impact in the instruments, and by manifest observable expressions. For one subject, we found a slight deviation in the instruments. These three subjects also reported self-rated FOG in the hedge opening with a white doorframe.

For subjects one and two, further tests were carried out. The tests showed that subject one and subject two differed noticeably. Subject number one had a clear FOG reaction. One could observe a more labored gait with him. He pulled up his shoulders and modified the landing of one foot. Subject number two had a general FOG reaction of 6.6 throughout the experiment with the doorframe, but only 1.6 when he passed through the hedge opening without a doorframe.

When the test subject wore a cylinder which limited his peripheral visual field—*i.e.*, when the person had limited capacity to visually fully interpret his environment [6,38], FOG reactions were triggered even when the subject passed through green hedge openings. During no other test occasion did any of the subjects have any FOG reaction when passing through hedge openings without the doorframe.

An unexpected but clear finding was that all subjects describe strategies they use to reduce the impact of FOG in everyday life. The observations confirm that some of the subjects used strategies to prevent/avoid FOG reactions and locking. Subjects 1 and 4 did not use any visible strategy during the experiment. Subject number two walked with an unchanged pace through all the tests. By observing, we could see that the subject used clear marching-steps to avoid locking. Subject number three was skipping along in the experiment setting, also with an unnatural gait. Subject number four told us of a type of a dancing walk he used when he experienced FOG reactions and lockups. He also sought to prevent FOG by using the strategy of shutting his eyes before and during the passage; nonetheless, he had a clear FOG reaction.

Subject number five estimated his experience of FOG as very strong when passing through the doorframe, but used a deliberate strategy of increasing his walking pace and thus affecting or delaying FOG reactions from being triggered. He has found ways to make his way forward by strategically choosing a different kind of gait to his normal gait. It is more like dancing or marching than walking, techniques that many people use to delay the onset of locking. He says he can experience FOG reactions at any time and has probably experienced a need to move forward in this way. He also succeeds in making his way through all openings without apparent problems. However, based on his self-assessments, this required a lot from him both mentally and physically. He needed to put a lot of effort and concentration to partly counteract his natural walking pattern.

Subject number one had a kind of walk which appeared to be his usual walking pattern. He did not try to counteract this walking pattern, and instead he had the strongest FOG reaction. Could it be that when he walked without trying to subdue or prevent a FOG reaction, his gait established an unconscious communication with the surrounding environment; a communication which, among other things, has to do with avoiding dangers of various kinds [59]?

What is clear is that the two who did not show any measurable effects from the accelerometer when walking through the doorframe, had no visible reactions from the waist down. The strategies they used were focused on their legs. Both, conducting the experiment without measurable results from the accelerometer, had a non-natural type of gait throughout the study. Such strategies were not mentioned in the study by Almeida and Lebold [11].

An additional finding must be highlighted: three of the subjects had a significant physical impact when they were low-medicated, which they also expressed in their interviews. This impact may lead to a perception of low motion control and may also cause concern for the individual: An inner awareness of an inability to cope with the situation physically may easily trigger a protective, survival reflex.

The clinical picture of Parkinson’s disease includes an array of common basic problems, but PD can manifest itself in different ways. The results of this study show that Parkinson’s symptoms and their physical and mental consequences are unique [6,15,41]. They vary depending on, for example, medication and stress levels [6,18]. Subjects described their experience of their illness and their individual experiences of cognitive and physical impact on low levels of medication. Some felt that the physical consequences were most apparent while others described psychological influences and effects as being the most dominant (see Table 1). There were also various ways individuals tried to prevent or regain control from a FOG reaction by, for example, marching, dancing, crawling, hopping using a walker, or changing their stride and step speed [60].

FOG has been interpreted as motor blockings, but recent research suggests that other factors may contribute. FOG has been shown to be associated with anxiety, depression, stress, and pain [4,6,14,16,61]. In interviews, some of the relatives expressed surprise that their family member did not have any FOG reaction or lockings when passing through the doorframe. They reflected on reasons why. For instance, one relative said that if the subject experiences something as fun; it has a clear positive impact, and reduces his FOG reactions. Another family member mentioned that the expectations of the results of the tests could reduce the subject’s propensity for FOG and may be a factor affecting the test results [6]. This can also happen during a doctor’s visit, when trying to but failing to trigger and exhibit a FOG reaction.

A doorframe in a hedge opening is not natural. It is an unnatural element in the outdoor environment. Three of the subjects had FOG reactions when passing through the doorframe—The visual information of the external, more natural environment, did not fit (straight lines and right angles) [6,38,62]. The subjects’ reactions were different, depending on whether the subjects were able to use a strategy to avoid locking or not. One may suspect that the visual experience of the doorframe triggered the FOG reactions. In all experiments where the signals from the environment were either difficult to interpret (doorframe) or where there were no signals of a safe environment (cylinder), a FOG reaction was triggered. Lacking a full overview of the environment and thus not being able to interpret it [38,62] may cause a hazard response [63]. A frequently used technique in horror films is the limiting of that the visual field of view. Appleton’s prospect/refuge theory [64] involves an overview of the place evoking safety and security. In addition, subject number two estimated big differences in his experience of general FOG in test rounds without doorframes compared with test rounds when passing through a doorframe (see Table 3). Could this difference in estimation of general FOG be an expression of that the constructed element—This doorframe—Affecting the interpretation of the environment: A “danger” signal is roused? From a survival standpoint, it is considered to better to hit the brakes once too often than once in a while [65,66].

### 4.2. Discussion, Related to Mechanisms of Information and Action

Decision making and action depend on us properly understanding and interpreting the information we take in. It is about paying attention to our surroundings, making decisions, and then acting. Attention Restoration Theory [37,67] argues that we have two types of attention, directed attention and involuntary attention. There are two ways for the brain to take in information: Bottom-up, when information comes to us from the environment and is then processed, and top-down, where one is willfully looking for information [68]. Directed attention is more about searching for information top-down, while involuntary attention is more about finding information bottom-up. Kaplan & Berman [68] develop ART by claiming that most activities are dependent on both involuntary attention and directed attention. A task that is initially more to be considered as using directed attention can—The more used you become to the activity, through practice—switches to an automated behavior and thus uses the more involuntary attention in its implementation. For example, when training, a high jumper works on automating processes. The strategies many of the subjects in this study demonstrated may be because they have practiced “unnatural” gait behaviors that do work, hence, they automatically fall back on them.

Kahneman [69] suggests that an individual’s decision making and action assumes that a solution is quick and easy (System 1). What usually works, but can sometimes lead to wrong conclusions. We also have a slow, reflective system (System 2), which we rarely use. Partly because System 2 is very slow, but also because it is very tiring to use. This can be interpreted as System 1 mainly using involuntary attention. System 2 can be interpreted as a special case of directed attention—An extreme directed attention, where nothing is based on routine or experience. Therefore, the subjects who practiced strategies in our study can make extensive use of System 1. Those who suddenly suffered from FOG and severe lockings tried to get out of this, maybe by using directed attention and System 2 in their decision-making, and therefore became greatly fatigued. We would like to discuss System 0, where decision-making is a pure reflex.

Work on nonhuman primates has shown that top-down attention is driven more by Pre-Frontal Cortex (PFC), whereas bottom-up attention is driven more by the Parietal-Temporal-Occipital region (PTO) [70]. The PTO area consists of an association-area of the cerebral cortex, where skin-muscle-joint-sensations along with visual and auditory information are coordinated as an “inside view”: It is a 3-dimensional map which “shows the brain” partly how the world is constructed on the basis of visual and auditory stimuli, and partly the body’s position and movement in this environment; this is particularly based on information from muscle- and tendon organs and from the joint capsules [70]. People with PD have trouble interpreting information from their body. They are more dependent on visual information than people normally tend to be, and are more reliant on peripheral visual feedback [71] and on impressions without ambiguity—Otherwise a need for focused attention occurs [38].

When peripheral vision was limited using a paper cylinder in the tests, it created a feeling of insecurity. The first FOG reaction occurred when passing through the hedge opening without the doorframe and with the cylinder. A clear “no danger” signal seems to be needed in order not to trigger a FOG reaction.

When one’s surroundings convey sensory input that signals “no danger”, this can contribute to the absence of FOG, like when passing through the hedge opening without the frame. Compared to an opening with a doorframe, a green hedge opening may also put less of a load on a person who always uses a technique to avoid FOG, as can be seen from subject number five, who experienced high self-rated FOG when he passed the doorframe in a hedge opening. Can built elements (straight, square, “non-natural”) trigger a “danger” reaction in us? A congenital ability in small children to avoid precipices on a surface consisting completely of straight lines and right angles has been demonstrated [72]. Is the FOG reaction triggered when the white doorframe was encountered analogous with this?

Although all subjects experienced FOG-reactions at home and in the 360 degree test before the trials, they experienced no FOG-reactions in their gait cycle during tests in the more “natural” environment without any built element. This can be interpreted as an influence of a natural outdoor environment inhibiting or not triggering a FOG reaction. Neuroscience describes how human behavior is generated by information processing, consciously and unconsciously, with a focus on managing life and, in this, mixing the present with memories. There are descriptions of how we continuously interpret and make not yet made conscious decisions in an on-going stream of impressions, from both internal and external stimuli [73]. This co-activation creates predictions of what is highly likely to be relevant in a specific situation. Our hypothesis is that what we see in this study are two different unconscious “predictions”: “danger” and “no danger”.

Perceiving certain animals (e.g., snakes and spiders), the sight of blood, heights, and darkness can quickly and unconsciously trigger a danger reaction in us [74]. The physical environment itself may also trigger similar reactions of anxiety and fear, as well as security and safety [31,75]. Several researchers describe the necessity of a non-threatening natural environment in order to lower individual people’s levels of stress [31,76]. The biophilia hypothesis supports these findings. The hypothesis suggests that human responses to the natural environment in its simplest form are about “love of life and living systems”: Attraction or a philic reaction. Unlike phobias; philias are about the positive feelings people have toward certain environments, activities and objects in their natural milieus [77,78]. An environment can signal a calm, positive, warm, interesting, and secure atmosphere, or the opposite—An insecure, threatening, and distressing atmosphere. These signals create communication [59] and a flow of emotional tones [79]. These tones can signal “danger” or “no danger”. This affects the prediction and the action. The emotional tone is a prelinguistic language, talking through our affects [59].

When surroundings are experienced as ominous or threatening, the “danger response” will be promptly switched on, triggering freezing: A reaction which, in its purest form, is about survival [74]. An “avoidance” reaction (flight or freeze) can take place without us consciously “seeing” the dangerous situation [80]. We assume this could be what we find in our Parkinson’s patients with FOG: A chain of simulated stimuli and responses that are activated, resulting in freezing, an avoidance reaction. When stressed, the individual relies more on affects [36,65,66]. Parkinson’s patients may possibly have a weakened ability to inhibit strong danger-reactions that may occur when passing through doorways. Visual impressions from nature signaling “no danger” have the ability to rapidly reduce stress via our affects [36,80], which may be crucial to persons with PD and FOG. Is FOG an oversensitive, unconscious avoidance reaction? 

What happens when the body freezes? The eyes capture something (bottom-up). It is processed in the brain and communicated to different systems. Öhman [74] speaks of four main categories of phobia, those to do with animals (snakes, spiders), social phobias, phobias of injury and blood, and spatial phobias (steep precipices, open spaces and confined spaces). We suggest that what is happening regarding FOG may have to do with spatial phobias. Our evolutionary history is obvious in the fears and phobias that we humans exhibit and readily learn. We are more likely to fear events and situations that were threats to the survival of our predecessors, such as potentially deadly predators, wide-open spaces and heights, than to fear the most commonly encountered potentially deadly objects in our modern environment, such as firearms or motorbikes [81,82].

Clearly, false negatives (that is, failing to bring about a defense to a potentially lethal stimulus) are more evolutionarily costly than false positives (that is, bringing about the response to a stimulus that in effect is risk-free). Thus, from an evolutionary perspective, it is likely that perceptual systems are biased toward discovering threat. To guarantee an effective defense when life is at stake, the system is biased to “playing it safe” by sometimes initiating a defense to something which, on closer examination, turns out to be harmless. Researchers like LeDoux [83] explain the freezing-response as an instinctive action that is carried out in the service of protecting an animal or human being. In our theory, freezing is a response that automatically occurs without cognitive evaluation or planning. From an evolutionary point of view, freezing, in a context of spatial phobia, is an absolutely correct reaction, so a person, e.g., does not fall off a cliff. Freezing may occur because the incoming stimulus has triggered an intense response reaction that momentarily inhibits access to any thinking and planning. Yet, as the intensity of the physiological response declines, access to planning and thinking can be reinstated.

Usually when a visual stimulus is detected in the environment, a signal goes to the thalamus, and then to the sensory cortex (the high road) where it is interpreted. But if the stimulus is interpreted as extra hazardous, signals are sent directly to the amygdala (the low road). From the amygdala, signals that can trigger a freeze reaction are sent to the midbrain. It is described as a “quick and dirty” transmission route. It “probably does not tell the amygdala much about the stimulus, certainly not much about Gestalt or object properties of the stimulus, but it at least informs the amygdala that the sensory receptors of a given modality have been activated and that a significant stimulus may be present” [84], so that the amygdala can start early activation of defense responses. This system is explicitly adopted to be adaptively biased toward false positives rather than false negatives. This is because it is less costly to abort falsely initialized defense responses than to fail to produce a defense when the threat is real [74].

## 5. Conclusions: A Need for a Biophilic Environment?

PD patients appear unusually clearly affected by a safe natural environment in a positive way. One may suspect that others with difficulties in interpreting and managing a physical environment in a similar manner (such as people with autism spectrum disorder, Asperger’s, ADHD, Alzheimer’s or the “oldest old”) can greatly benefit from what we are proposing to call a biophilic environment. Studies have shown that people in life crises also have difficulties managing their environment [59], which has led to a theory of “Supportive Environments”, illustrated by a pyramid.

**Figure 3:**
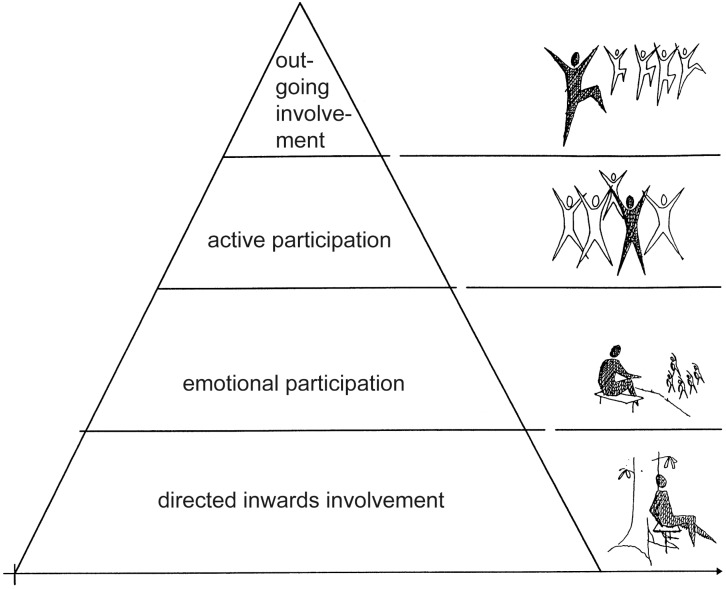
Pyramid of supporting environments and levels of function and coping abilities. From Ottosson and Grahn [85] The illustration is reproduced with permission from the authors.

Ottosson and Grahn [85] present a model, a pyramid, in which the physical and social environment is related to the individual’s executive function. A person’s executive function is his/her capacity to see information, sort information, prioritize, plan, and carry out a duty [86]. The y-axis shows their executive function and the x-axis their degree of sensitivity to their environment. People have different abilities to function with regard to their environment depending on the status of their executive function at the time. The same individual may need supporting environments on different levels of the pyramid at different times. The amount of executive function determines the highest level in the pyramid of situations with which people can cope (see Figure 3). At the bottom of the pyramid, people have a need for a supporting environment consisting of environments that can be interpreted as safe quite reflexively. This need is, as we interpret it, subconscious, and a necessity for functioning. Given somewhat more mental/physical capacity, the individual may wish to find other, more complex environments, containing more built elements. At the top of the pyramid, mental and physical capacity is strong. Referring to this pyramid, we suggest that individuals who have a low capacity to interpret and manage their environment have an immediate need for a biophilic environment—A necessity of biophilic environments is that they can act on reflex or “System 0”.

The main finding of this study is that the physical environment appears to affect function in people with PD. The design of visual environments could thus be of special importance where people with PD live, work, are cared for, and rehabilitated. Patients with PD and FOG could get a simpler life by consciously prioritizing stays in natural surroundings [41]. However, many other groups of people could also benefit from this, without our being aware of it [80]. An important cause of falls in the elderly in general is believed to be that their perception skills have changed with the years, and possibly communication between perception and motor skills [87]. For example, strong color contrasts on the floor can be misinterpreted and lead to falls. It has been shown that impaired vision combined with slow reaction times and swaying explains 75% of all falls in older people [88]. Freezing of Gait may be a common cause of falls in the elderly. Consequently, subconscious reactions regarding the shape and contents of surrounding physical environments, which significantly affect behavior and health, may apply to many groups of people. Many fall injuries could therefore be prevented by measures in construction, both indoors and outdoors. This study focuses on FOG in older people with Parkinson’s disease. Five cases are studied carefully, regarding how the environment can influence whether FOG is triggered or not. The results suggest that the design of the environment has an impact on how it is interpreted, instinctively, by the Parkinson’s patients, and if FOG is triggered or not. As individuals, people with Parkinson’s Disease are very different from each other in terms of both symptoms and function. The strength of this study is that we had a well-defined group who gave us the confidence to explore the subjects using many methods. Each subject was seen as a case that could be followed separately, while they could also be compared with the other cases in the study. The different methods of measuring FOG showed similarities to a great extent. Self-estimates of general FOG showed that all subjects experienced FOG during the test situation, and they handled this situation in different ways. Accelerometer measurements, self-estimates, interviews and observations gave a consistent picture. We seek to explain the results obtained in this study using environmental psychological theories such as the biophilia hypothesis, and wordless communication related to danger/non-danger.

However, there is no doubt that this study contains few cases and, therefore, the results need to be reproduced to confirm this study’s results and the issues put forward in this paper. The results may come to have significance in, among other things, the design of residences, workplaces, and rehabilitation/hospital environments. The results give rise to ideas for future studies, where indoor environments should be highlighted on the same theoretical foundation. For example, whether wider doors make a difference with respect to function in people with PD, or whether other forms of doors, special floor patterns, open floor plans, larger windows opening on to outdoor vegetation and/or more plants indoors have an impact.

Our results, if repeated in future studies, may have great significance to the everyday lives of PD patients as well as elderly people in general. Further studies may show whether environments can be built so that fewer older people are subjected to FOG and fall injuries.
